# NAT10‐mediated RNA ac4C acetylation contributes to the myocardial infarction‐induced cardiac fibrosis

**DOI:** 10.1111/jcmm.70141

**Published:** 2024-11-01

**Authors:** Jun Li, Feierkaiti Yushanjiang, Zhao Fang, Wan‐li Liu

**Affiliations:** ^1^ Department of Cardiology Renmin Hospital of Wuhan University Wuhan Hubei China; ^2^ Cardiovascular Research Institute Wuhan University Wuhan Hubei China; ^3^ Hubei Key Laboratory of Cardiology Wuhan Hubei China; ^4^ Department of Pediatric, Maternal and Child Health Hospital of Hubei Province, Tongji Medical College Huazhong University of Science and Technology Wuhan Hubei China

**Keywords:** ac4C acetylation, apoptosis, cardiac fibrosis, myocardial infarction, NAT10

## Abstract

Cardiac fibrosis is featured cardiac fibroblast activation and extracellular matrix accumulation. Ac4C acetylation is an important epigenetic regulation of RNAs that has been recently discovered, and it is solely carried out by NAT10, the exclusive enzyme used for the modification. However, the potential regulatory mechanisms of ac4C acetylation in myocardial fibrosis following myocardial infarction remain poorly understood. In our study, we activated fibroblasts in vitro using TGF‐β1 (20 ng/mL), followed by establishing a myocardial infarction mouse model to evaluate the impact of NAT10 on collagen synthesis and cardiac fibroblast proliferation. We utilized a NAT10 inhibitor, Remodelin, to attenuate the acetylation capacity of NAT10. In the cardiac fibrosis tissues of chronic myocardial infarction mice and cultured cardiac fibroblasts (CFs) in response to TGF‐β1 treatment, there was an elevation in the levels of NAT10 expression. This increase facilitated proliferation, the accumulation of collagens, as well as fibroblast‐to‐myofibroblast transition. Through the administration of Remodelin, we effectively reduced cardiac fibrosis in myocardial infarction mice by inhibiting NAT10's ability to acetylate mRNA. Inhibition of NAT10 resulted in changes in collagen‐related gene expression and ac4C acetylation levels. Mechanistically, we found that NAT10 upregulates the acetylation modification of BCL‐XL mRNA and enhances the stability of BCL‐XL mRNA, thereby upregulating its protein expression, inhibiting the activation of Caspase3 and blocking the apoptosis of CFs. Therefore, the crucial involvement of NAT10‐mediated ac4C acetylation is significant in the cardiac fibrosis progression, affording promising molecular targets for the treatment of fibrosis and relevant cardiac diseases.

## 1 INTRODUCTION

Myocardial infarction (MI) is a prevalent cardiovascular occurrence and a significant factor in the development of heart failure (HF).^1^ The obstruction of coronary flow results in a lack of oxygen and nutrients in the impacted regions of the heart, leading to the death of cardiomyocytes (CMs). Subsequent to the loss of CMs, an inflammatory reaction takes place, followed by the creation of granulation tissue containing newly generated microvascular structures and extracellular matrix‐producing cardiac fibroblasts (CFs).^2^, ^3^ Ultimately, the excessive deposition of the extracellular matrix can lead to stiffness in the myocardium, resulting in diastolic dysfunction, or impact the entirety of the left ventricle, causing dilatation and systolic dysfunction, ultimately culminating in heart failure and mortality.^4^ Therefore, how to effectively reduce myocardial interstitial fibrosis is an important strategy to prevent and treat heart failure after MI.

Epitranscriptomic RNA modifications play a significant role in regulating gene expression and impacting various cellular and biological functions.^5^, ^6^ Among these modifications, ac4C acetylation has been shown to enhance mRNA translation and stability within cells.^7^, ^8^ N‐acetyltransferase 10 (NAT10) is the sole enzyme responsible for catalysing the process of RNA acetylation modification, and its dysregulation has been implicated in the pathogenesis of various diseases.^9^, ^10^, ^11^ Recently, Wang et al. identified the heart‐apoptosis‐associated piRNA (HAAPIR), which interacts with NAT10 in a direct way and advances ac4C acetylation of the Tfec mRNA transcript and then elevates Tfec expression. TFEC can subsequently enhance the BCL2‐interacting killer (Bik) transcription, which leads to the Bik accumulation and cardiomyocyte apoptosis progression in ischemia heart diseases.^12^ However, it remains to be elucidated whether NAT10 regulates myocardial interstitial fibrosis in ischemia heart diseases as an mRNA acetylation regulator.

## 2 MATERIALS AND METHODS

### 2.1 Establishment of a mouse model of MI

All experimental procedures involving animals complied with the guidelines for the Care and Use of Laboratory Animals and were approved by the Committee on the Use and Care of Experimental Animals of Renmin Hospital of Wuhan University (WDRM‐20220104B). The ARRIVE 2.0 guidelines were followed for animal experiments. Male 8‐week‐old C57BL/6 mice were supplied by the Laboratory Animal Center of Renmin Hospital of Wuhan University. The mice were grouped according to a random number table into either sham surgery or a MI group with or without the NAT10 inhibitor, remodelin treatment. The animals were administered anaesthesia intraperitoneally injection of 3% sodium pentobarbital at 40 mg/kg and ventilated with an animal ventilator. A thoracotomy was performed on the left side through the fourth intercostal space, resulting in the exposure of the heart by opening the pericardium. Myocardial infarction was induced by ligating the left descending coronary artery (LAD) using a 7/0 nylon suture positioned 2 mm below the demarcation line between the left atrium and ventricle. Notable elevation of the S‐T segment in electrocardiographic recordings confirmed the presence of myocardial ischemia. Similar to the MI group, the sham‐operated mice experienced the same experimental procedures without ligation of LAD. According to a previous study,^13^ remodelin (2 mg/kg) was dissolved in DMSO and administered to the animals by i.p. injection every 2 days for 4 weeks. At 4 weeks after MI, the mice were subjected to euthanasia by cervical dislocation.

### 2.2 Mouse neonatal CFs isolation and culture

Neonatal male mice were utilized for the isolation of CFs (1–3 days). The collected myocardial tissue was subjected to enzymatical digestion with 0.125% collagenase type II and 0.25% trypsin–EDTA. After 90 min of cardiomyocyte adhesion, CFs were isolated and cultivated in fresh complete DMEM encompassing 10% fetal bovine serum, along with a 1% penicillin and streptomycin mixture. The process took place at 37°C in a 5% CO2 incubator. Subsequent experiments were carried out through the second and third generations of CFs. After starving for 24 h in serum‐free medium, TGF‐β1 (20 ng/mL; available from Peprotech, Rocky Hill, NJ, USA) with or without Remodelin (20 μM) was appended into culture plates for 36 h upon multiple experiment treatments as in the previous study.^14^ The CFs were subsequently acquired for further assays.

### 2.3 Histopathology

Hearts of each group were excised and fixed in 4% formalin, followed by embedding in paraffin, sectioning into 5 μm slices, as well as subjected to conventional haematoxylin–eosin staining according to our previous study.^15^ The Leica Microsystems microscope (Germany) was utilized for the analysis of the sections. Masson trichrome staining was performed simultaneously. Collagen staining was performed on the heart sections according to the Masson trichrome stain to assess the degree of fibrosis. Finally, with the Image‐Pro‐Plus 6.0 software (Media Cybernetics, Inc., MD), the fibrotic areas were examined under the microscope.

### 2.4 Quantitative reverse transcription PCR (qPCR)

The synthesis of cDNA utilized the RT First Strand cDNA Synthesis Kit (Servicebio, China) post the total RNA extraction (RNAiso Plus, TaKaRa, Japan). SYBR Green qPCR Master Mix Kit (Low ROX) (available from Servicebio, China) combined with the ABI ViiA7 Real‐Time PCR System (available from Applied Biosystems, USA) was utilized for RT‐qPCR.

### 2.5 Western blot assay

The isolation of the protein was achieved with SDS‐PAGE, which was transferred onto a PVDF membrane and blocked with 5% non‐fat milk. The membrane was cultivated with collagen I (Col I, 1:1000, ab270993, Abcam), collagen III (Col III, 1:1000, Proteintech, 22,734‐1‐ap), α‐SMA (1:1000, ab21027, Abcam), fibronectin (1:1000, ab2413, Abcam), NAT10 (1:1000, ab194297, Abcam) and GAPDH (1:1000, ab9485, Abcam), followed by cultivation with horseradish peroxidase (HRP)‐conjugated secondary antibodies. To visualize and quantify the protein bands, a densitometer from ImageJ software (NIH, Bethesda, MD) was utilized for measurement.

### 2.6 Cell proliferation assay

As previously described, a Cell Counting Kit‐8 (CCK‐8, Dojindo, Tokyo, Japan) assay was implemented for measuring cell proliferation.^16^ In short, cells were subjected to seeding in a 96‐well plate at 1 × 10^3^ cells/well and cultivated for the indicated time points (0, 12, 24, 48 and 72 h). The absorbance at 450 nm was estimated after 1‐hour incubation with 10 μL of CCK‐8 reagent.

### 2.7 Immunofluorescence staining

CFs were cultured in confocal dishes and allowed to attach to the walls of the dishes, with subsequent 4% paraformaldehyde fixation for 20 min. This was followed by the use of 0.25% Triton‐X 100 to cause rupturing of the cell membrane. The 5% bovine serum albumin was used for blocking for 1 h. Afterward, cells were co‐cultured overnight with primary antibodies (NAT10 and anti‐ac4C antibody) at 4°C, followed by 1‐h cultivation with the corresponding secondary antibodies (goat anti‐rabbit DyLight 405 conjugated, 1:200, Rockland 611–146‐002; goat anti‐mouse DyLight 549 conjugated, 1:500, Thermo Fisher A28175) the next day at ambient temperature. The 4′,6‐diamidino‐2‐phenylindole‐stained nuclei (10 min) were pictured under a laser confocal microscope.

### 2.8 Immunohistochemistry (IHC) analysis

Heart myocardial tissues were subjected to isolation and fixation with 4% paraformaldehyde, as well as incubation with primary antibodies against NAT10 and anti‐ac4C antibodies. The following day, the samples were exposed to secondary antibodies and stained with diaminobenzidine per the directions specified by the kit (ZSGB‐Bio, China). ImageProPlus 500.6 (Media Cybernetics, USA) was used for data analysis.

### 2.9 Quantification of ac4C in total RNA

Upon harvesting the total RNA from each sample, a Total ac4C Acetylation Quantification Kit (GK‐4040, Colorimetric, available from Genelily Biotech, Shanghai, China) was utilized for the quantification of the ac4C levels. Briefly, 200 ng RNAs were first coated on assay wells before separately adding capture antibody solution together with detection antibody solution to every assay well in an appropriate diluted concentration. The colorimetrical quantification of the ac4C levels was performed via reading the absorbance at 450 nm wavelength, and then calculations were implemented following the standard curve.

### 2.10 Dot blotting

Total RNA from each sample was heated to 75°C for 5 min before cooling for 1 min and loading onto 0.45 μm Amersham Hybond‐N+ membranes (YA1760, from Solarbio, Beijing, China). After that, the membranes were cross‐linked and blocked, followed by overnight cultivation with an anti‐ac4C antibody at 4°C and 1‐h cultivation with an HRP‐labelled secondary anti‐rabbit IgG at 25°C. Subsequently, the membranes were utilized to visualize the proteins of interest using the chemiluminescent HRP substrate (WBKLS0500, from Millipore, Billerica, MA, USA). After that, the membranes upon exposure were dyed for 30 min with methylene blue staining buffer and then rinsed with ribonuclease‐free water. Lastly, the input RNA was scanned for evaluating its total content.

### 2.11 Flow cytometry

Cell apoptosis was further detected by flow cytometry (BD, FACS AriaIII) using the PE Annexin V Apoptosis Detection Kit (C1062M, from Beyotime, Nanjing, China) according to the manufacturer's recommendation. Briefly, Cells from each group were collected, resuspended in 500 μL binding buffer, and labelled with Annexin V‐FITC and propidium iodide (PI). The labelled cells were then incubated for 15 min in the dark. All samples were analysed using flow cytometry.

### 2.12 TUNEL staining

Apoptosis rates in cultured CFs were analysed by TUNEL staining using a terminal deoxynucleotidyl transferase (TdT) dUTP nick‐end labeling (TUNEL) assay (Beyotime, Nanjing, China) according to the previous studies.^17^, ^18^ In summary, the slides were incubated with TUNEL reaction mixture, apoptotic cells were labelled, and the total number of cells was determined by DAPI staining. The slides were viewed under a laser confocal microscope.

### 2.13 Acetylated RNA immunoprecipitation (acRIP)

The extracted total RNA was randomly fragmented, incubated with anti‐ac4C antibody (ab253039, 1:50, Abcam) to magnetic beads, purified, and analysed by RT‐qPCR.

### 2.14 Luciferase reporter assay

The BCL‐XL promoter or the wild‐type and mutant versions of BCL‐XL were cloned into the reporter plasmid. The luciferase activity was assayed using the Dual‐Luciferase reporter kit (Promega, USA) according to the manufacturer's instructions. Upon modulation of NAT10, either with or without Remodelin, the luminescence intensities of both firefly and Renilla luciferase were quantified, and the data were normalized against Renilla fluorescence.

### 2.15 RNA decay assay

We performed the actinomycin D assay to assess the stability of the mRNA. CFs treated with or without Remodelin or TGF‐β1 were incubated with actinomycin D (5 μg/mL, HY‐17559, MedChemExpress) for 0, 1, 3 and 6 h in 6‐well plates. The BCL‐XL mRNA half‐life was estimated by linear regression analysis of total RNA collected using the method described above for quantitative real‐time PCR analysis.

### 2.16 Statistical analysis

All the statistical analyses were employed with the application of GraphPad Prism 9.0 (GraphPad Software, Inc., San Diego, CA, USA). The data were depicted as the mean ± standard deviation. Each experiment was repeated in triplicate. A student's two‐tailed *t*‐test or one‐way analysis of variance (ANOVA) was utilized to discern between‐group variance with a significance level of *p*‐value <0.05.

## 3 RESULTS

### 3.1 NAT10‐mediated RNA ac4C acetylation is significantly elevated in cardiac fibrosis

**FIGURE 1:**
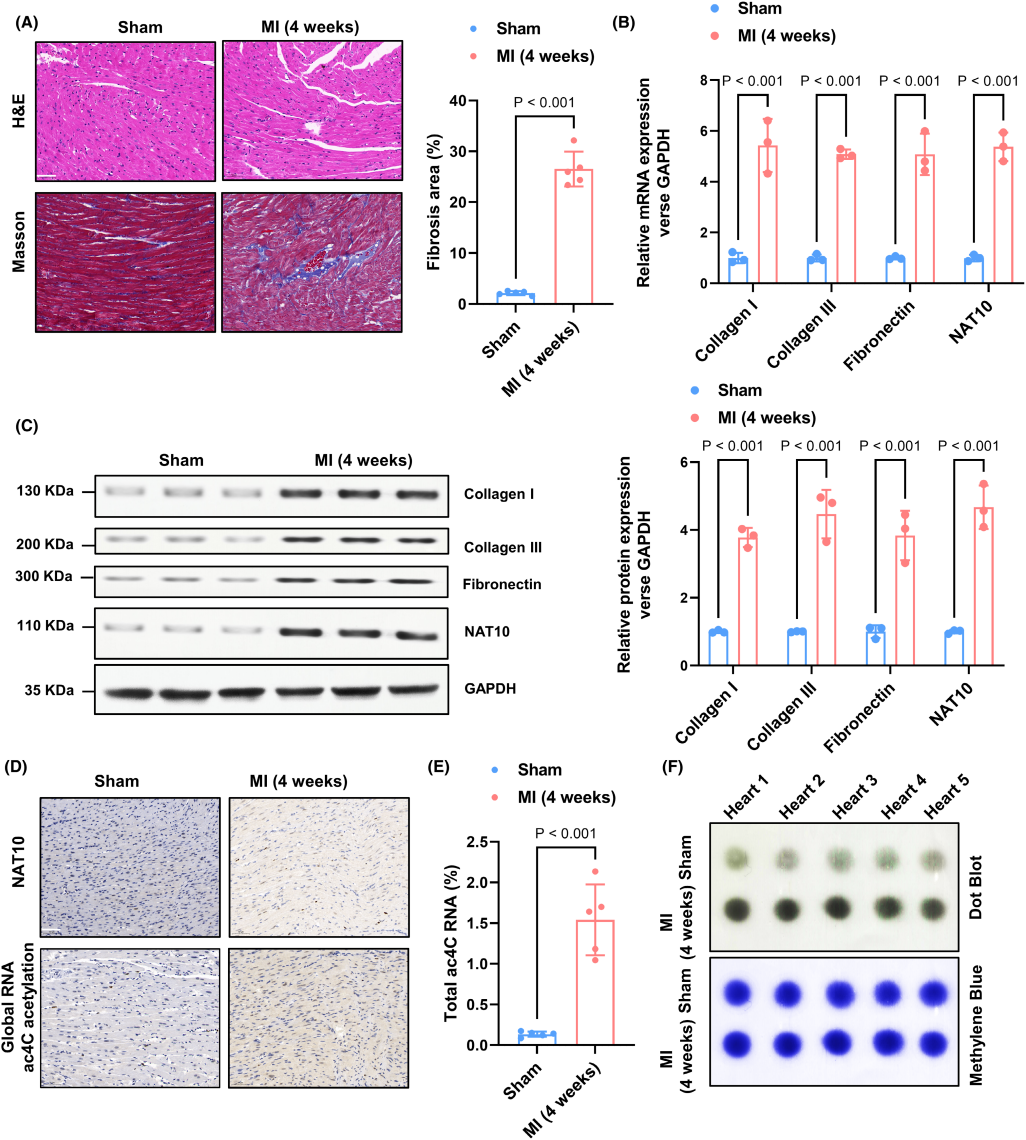
NAT10‐mediated RNA ac4C acetylation is markedly elevated in cardiac fibrosis. (A) Representative haematoxylin–eosin staining and Masson's Trichrome staining of the left ventricle 4 weeks after MI (scale bar: 50 μm) and the total fibrotic area quantification with Image‐Pro‐Plus 6.0. *n* = 5. (B) Col I, fibronectin, Col III and NAT10 mRNA levels were examined by RT‐qPCR assay. *n* = 3. (C) Proteins were isolated from each sample; subsequently, the western blot analysis was implemented for analysing the fibrosis‐associated protein and NAT10 levels. *n* = 3. (D) The expression of NAT10 and level of RNA ac4C acetylation were tested by IHC. Scale bar, 50 μm. (E, F) Extracted mRNA was detected by ac4C colorimetric quantification and dot blot assays. *n* = 5.

To investigate the potential involvement of RNA ac4C acetylation in the development of cardiac fibrosis, a mouse model of cardiac fibrosis was established by ligating the left anterior descending artery (LAD) for a duration of 4 weeks. The results showed an increase in interstitial fibrotic area and collagen accumulation, along with elevated levels of Col I, Fibronectin, and Col III in the myocardial infarction (MI) group (Figure 1A–C). Additionally, a significant upregulation of NAT10 expression was observed in the fibrotic tissue of the mice (Figure 1B,C). Immunohistochemical staining revealed a marked increase in NAT10 protein levels and ac4C acetylation in the MI mice (Figure 1D). The acetylation levels of ac4C were assessed through the utilization of the colorimetric method and dot blotting assay. The findings indicated a higher overall presence of ac4C in the MI group compared to the Sham group (Figure 1E,F). In conclusion, these outcomes provide evidence for the association between NAT10‐mediated RNA ac4C acetylation and the progression of cardiac fibrosis.

### 3.2 NAT10‐mediated RNA ac4C acetylation is significantly elevated in TGF‐β1 induced cardiac fibroblast proliferation and myofibroblasts

**FIGURE 2:**
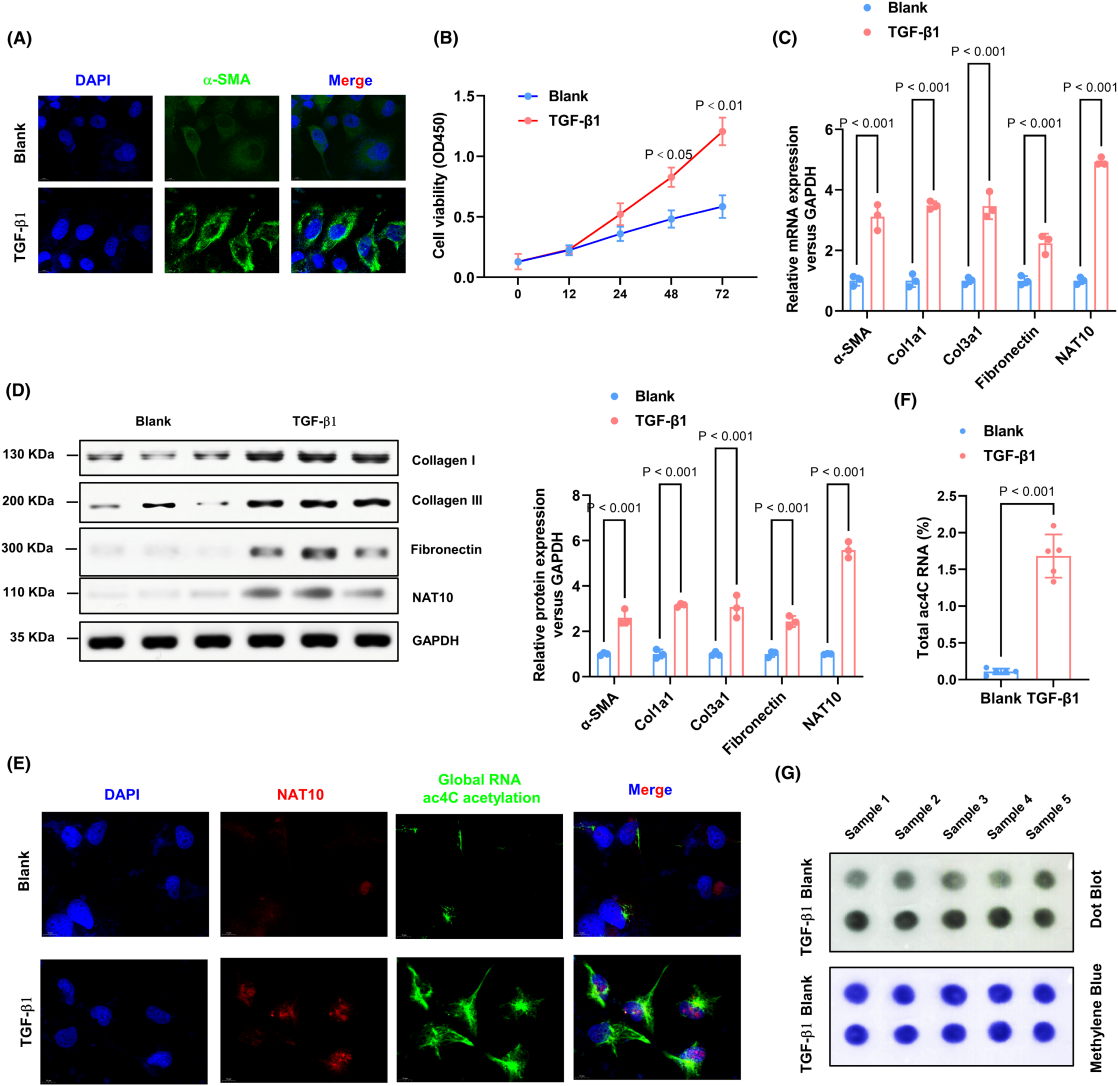
NAT10‐mediated RNA ac4C acetylation is significantly elevated in TGF‐β1‐induced cardiac fibroblast proliferation and myofibroblasts. (A) Immunofluorescence assays were employed to estimate α‐SMA levels in CFs, and nuclei were dyed with DAPI. Scale bar, 10 μm. (B) Cell proliferation was processed with CCK8 assay. *n* = 3. (C) Col I, fibronectin, Col III and NAT10 mRNA levels in CFs were assessed by RT‐qPCR assay. *n* = 3. (D) The fibrosis‐associated protein and NAT10 levels in CFs were tested by western blot analysis. *n* = 3. (E) Immunofluorescence assays were implemented to examine NAT10 expression and RNA ac4C acetylation. Red, NAT10; green, ac4C acetylation; blue, nuclei. Scale bar, 10 μm. (F, G) extracted mRNA from each group CFs was estimated by ac4C colorimetric quantification combined with dot blot assays. *n* = 5.

The activation of endogenous TGF‐β1 during the cardiac fibrosis process in response to myocardial infarction is evident. Enhanced α‐SMA protein expression and reduced cellular activity suggest the establishment of fibrotic models in cardiac fibroblasts following stimulation with TGF‐β1 (Figure 2A,B). Subsequent to the in vivo findings, the levels of profibrogenic proteins (Collagen I, Fibronectin, and Collagen III), as well as NAT10, were significantly increased following stimulation of cardiac fibroblasts by TGF‐β1 (Figure 2C,D). The immunofluorescence assay conducted on cardiac fibroblasts using specific antibodies revealed an upregulation of NAT10 and global RNA ac4C acetylation in the TGF‐β1 group (Figure 2E). The levels of ac4C mRNA were further assessed using colorimetric methods and mRNA dot blotting, showing a significant increase in ac4C levels in mRNA extracted from the TGF‐β1 group (Figure 2F,G). These findings suggest that alterations in NAT10‐mediated RNA ac4C acetylation may contribute to the proliferation of cardiac fibroblasts and myofibroblasts induced by TGF‐β1.

### 3.3 Inhibition of NAT10‐mediated RNA ac4C acetylation significantly impaired the TGF‐β1‐induced cardiac fibroblasts proliferation and myofibroblasts

**FIGURE 3:**
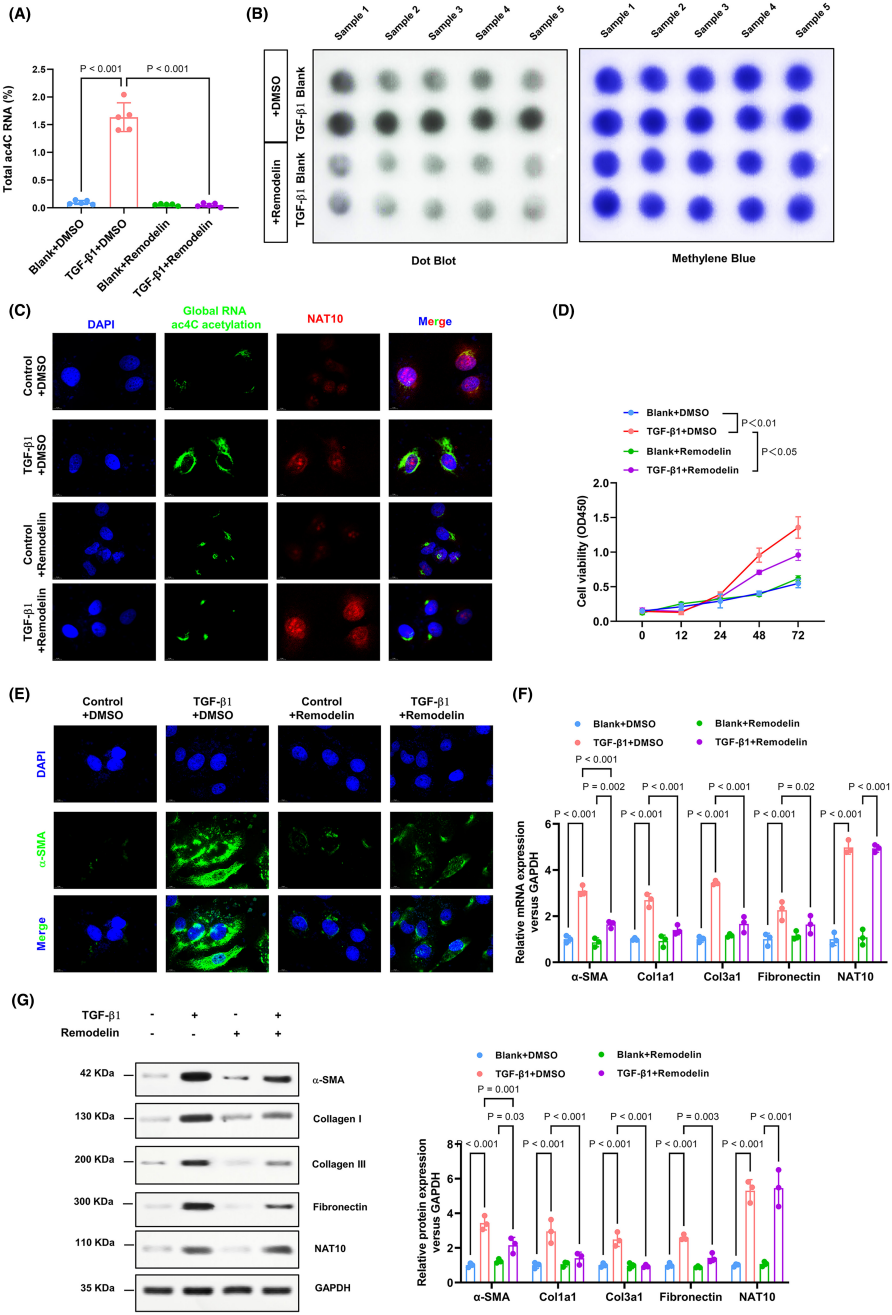
Inhibition of NAT10‐mediated RNA ac4C acetylation significantly impaired the TGF‐β1‐evoked CFs proliferation and myofibroblasts. (A, B) extracted mRNA from each group CFs was tested by ac4C colorimetric quantification combined with dot blot assays. *n* = 5. (C) Immunofluorescence assays were employed to evaluate NAT10 expression and RNA ac4C acetylation. Red, NAT10; green, ac4C acetylation; blue, nuclei. Scale bar, 10 μm. (D) Cell proliferation of each group was processed with CCK8 assay. *n* = 3. (E) α‐SMA expression in each group of CFs was assessed by immunofluorescence assays, and nuclei were dyed with DAPI. Scale bar, 10 μm. (F, G) the western blot combined with RT‐qPCR assay was adopted to analyze the fibrosis‐associated protein and NAT10 levels in CFs. *n* = 3.

Remodelin, as a chemical inhibitor of NAT10, was utilized to test its inhibitory impact on NAT10 in vitro.^10^, ^19^ NAT10 inhibited significantly abolished TGF‐β1‐induced global RNA ac4C acetylation (Figure 3A,B). Additionally, immunofluorescence analysis indicated an increase in global RNA ac4C acetylation levels in CFs exposed to TGF‐β1, which could potentially be counteracted by NAT10 inhibition (Figure 3C). Similarly, the assessment of cell viability using the MTT assay demonstrated that TGF‐β1 induction resulted in a decrease in cell viability, which was restored to approximately normal levels following treatment with NAT10 inhibition (Figure 3D). Subsequently, we investigated the potential of NAT10 inhibition to counteract the activation of cardiac fibroblasts. Through the use of immunofluorescence assays, we observed a significant reduction in α‐SMA expression in TGF‐β1‐induced cardiac fibroblasts following treatment with NAT10 inhibition (Figure 3E). Consistently, after remodelin treatment, the profibrogenic proteins (Col I, Fibronectin, and Col III) and their mRNA levels that increased in TGF‐β1‐induced CFs were significantly weakened (Figure 3F,G). To conclude, these findings unravel that NAT10 inhibition NAT10‐mediated RNA ac4C acetylation significantly impaired the TGF‐β1 induced cardiac fibroblasts proliferation and myofibroblasts.

### 3.4 Inhibition of NAT10‐mediated RNA ac4C acetylation significantly attenuated the MI‐induced cardiac fibrosis

**FIGURE 4:**
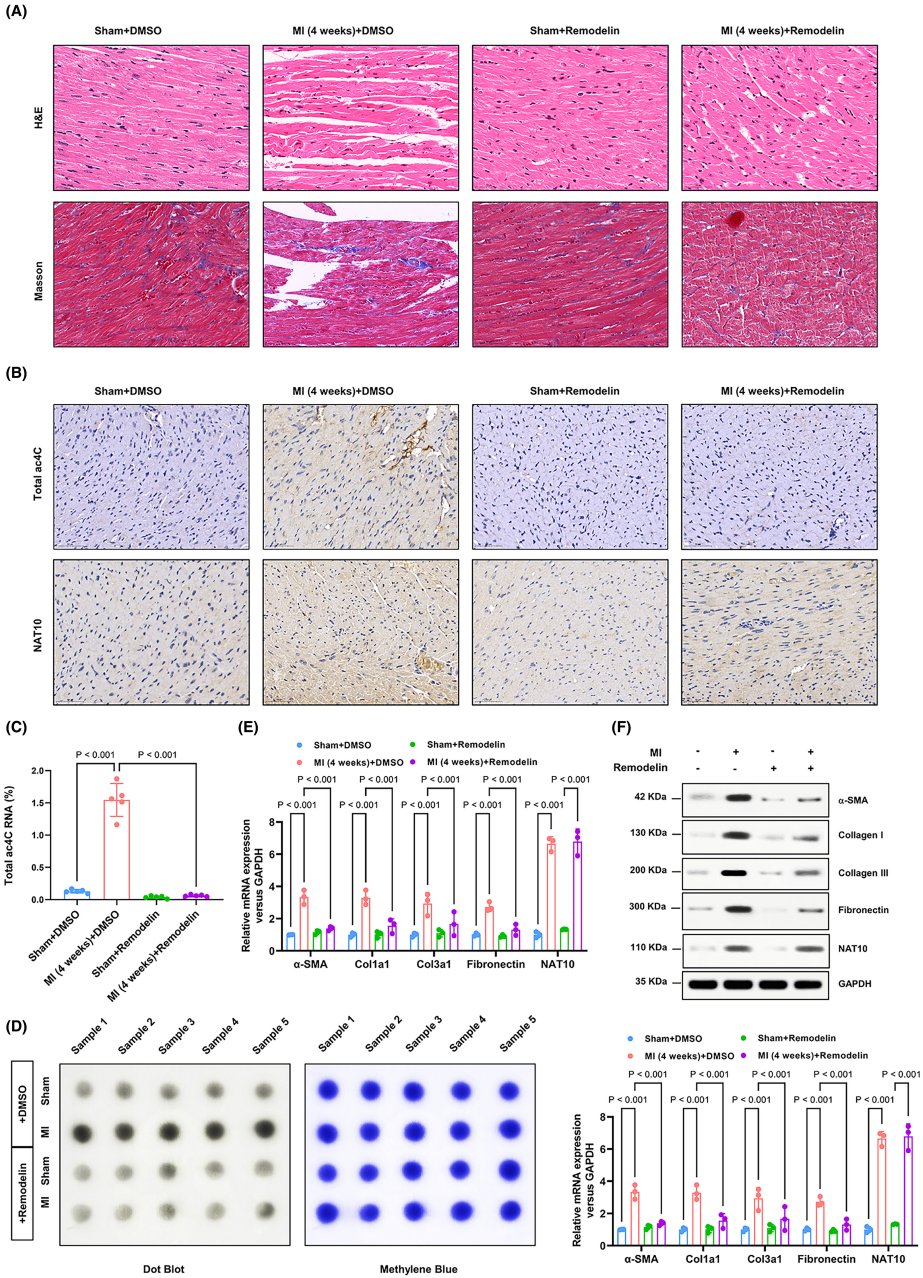
Suppression of NAT10‐mediated RNA ac4C acetylation significantly attenuated MI‐induced cardiac fibrosis. (A) Mouse heart sections from each group were dyed by H&E staining and Masson's trichrome staining. Scale bar, 50 μm. (B) The expression of NAT10 and level of RNA ac4C acetylation of every group was examined by IHC. Scale bar, 50 μm. (C, D) extracted mRNA from each group was detected by ac4C colorimetric quantification combined with dot blot assays. *n* = 5. (E, F) The western blot combined with RTqPCR assay was performed to analyse the fibrosis‐related protein and NAT10 expression in every group. *n* = 3.

In order to elucidate the pathophysiological role of NAT10 in post‐myocardial infarction (MI) cardiac fibrosis, we investigated the potential involvement of NAT10 in MI‐induced cardiac fibrosis using an animal model. Remarkably, inhibition of NAT10 resulted in reduced cardiomyocyte injury and collagen deposition in an MI‐induced mouse model of cardiac fibrosis, as demonstrated by histological analyses using haematoxylin and eosin (HE) and Masson's trichrome staining (Figure 4A). Furthermore, immunohistochemistry (IHC) and dot blotting analyses demonstrated that the inhibition of NAT10 resulted in the suppression of myocardial infarction (MI)‐induced upregulation of ac4C acetylation, while NAT10 expression remained unaffected (Figure 4B–D). Conversely, compared to the control group, MI led to an increase in profibrogenic proteins (Collagen I, Fibronectin, and Collagen III) levels, which was predominantly reversed by NAT10 inhibition (Figure 4E–G). In conclusion, these findings highlight the significant role of NAT10 inhibition as a key regulator of cardiac fibrosis through the enhancement of RNA ac4C acetylation.

### 3.5 Inhibition of NAT10‐mediated RNA ac4C acetylation significantly triggers the apoptosis of cardiac fibroblast

**FIGURE 5:**
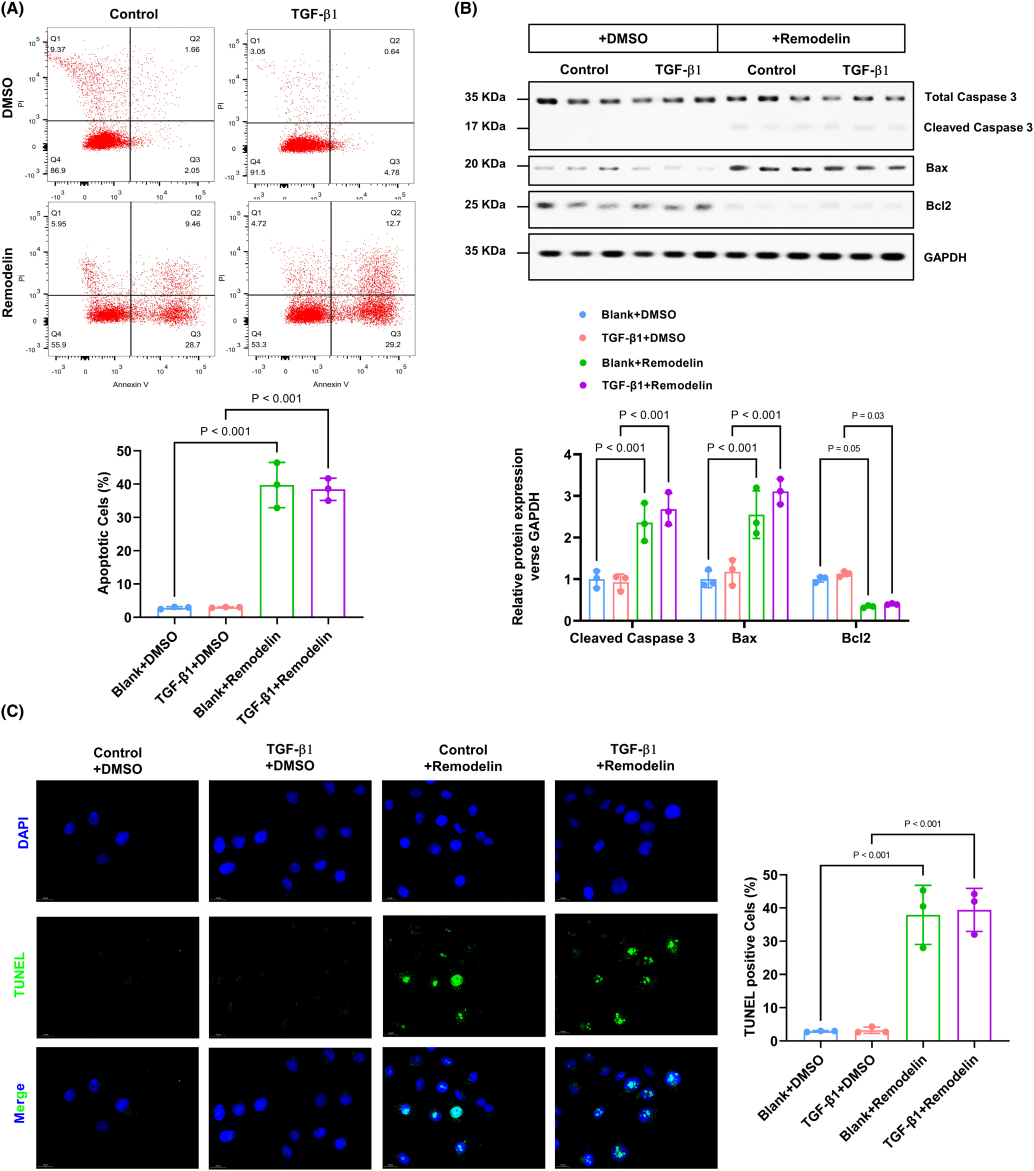
Inhibition of NAT10‐mediated RNA ac4C acetylation significantly triggers the apoptosis of cardiac fibroblast. (A) CFs were labelled with Annexin V‐FITC and propidium iodide (PI) and analysed by flow cytometry. *n* = 3. (B) The western blot assay was performed to analyse the apoptosis‐related protein in each group. *n* = 3. (C) TUNEL staining and the quantitative results in each group. Green, TUNEL‐positive nuclei; blue, DAPI. *n* = 3.

In order to assess the impact of NAT10 inhibition on CFs apoptosis, flow cytometry and TUNEL staining were conducted to analyse apoptotic alterations. The findings revealed no significant difference in apoptosis rates between the control and TGF‐β1 groups, whereas a notable increase in apoptosis rate was observed in cells treated with remodelin (Figure 5A). This outcome was further supported by TUNEL staining results (Figure 5C). Apoptosis‐promoting effects of NAT10 inhibition were further confirmed by western blot results showing that remodelin increased the expression of BAX, C‐Caspase3 and decreased the BCL‐2 level (Figure 5B). All these results implied that inhibition of NAT10‐mediated RNA ac4C acetylation significantly triggers the apoptosis of CFs.

### 3.6 NAT10‐mediated RNA ac4C acetylation significantly improves the stability of BCX‐XL mRNA and increased its expression in CFs

**FIGURE 6:**
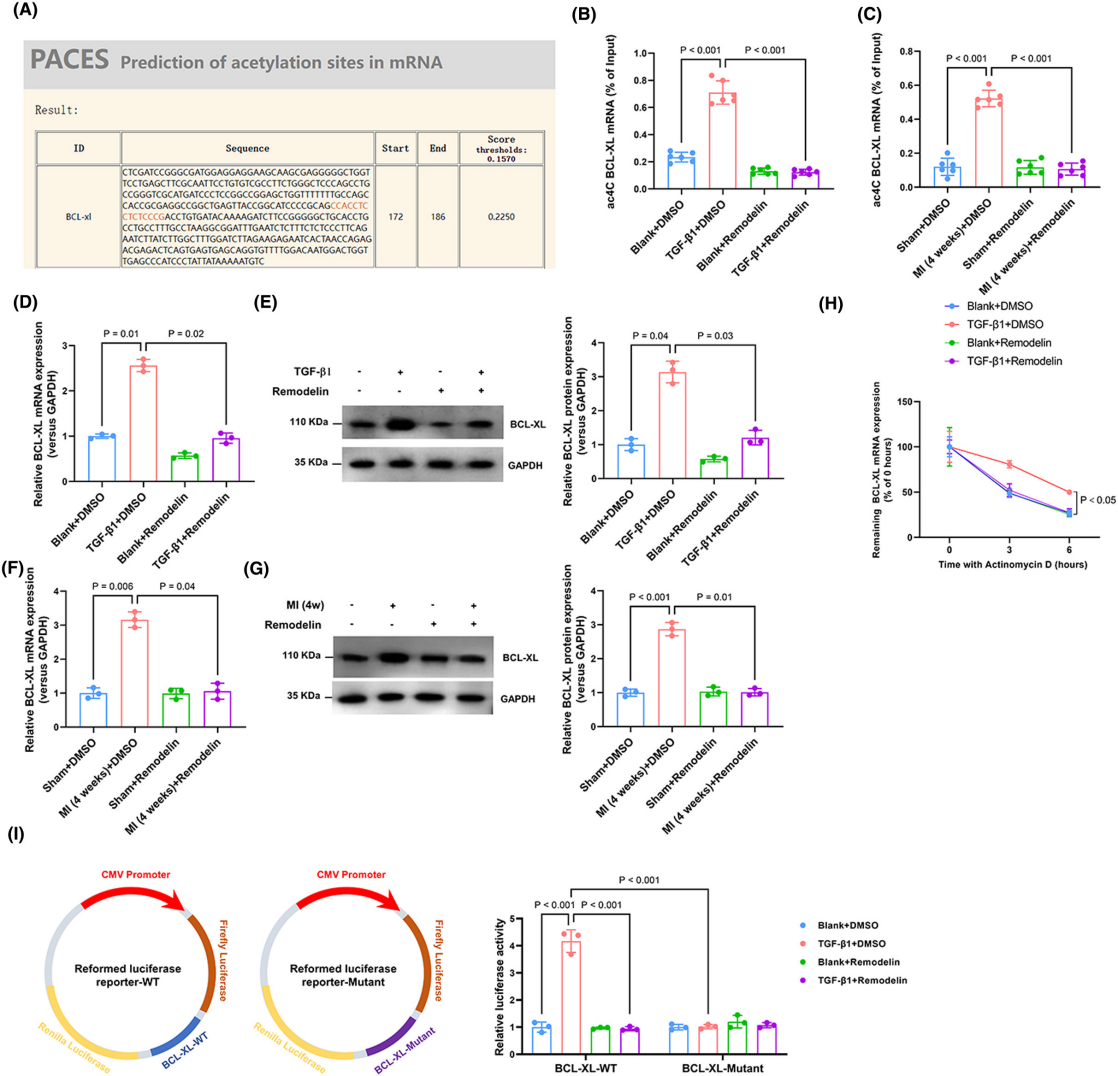
NAT10‐mediated RNA ac4C acetylation significantly improves the stability of BCX‐XL mRNA and increases its. (A) PACES tools (http://rnanut.net/paces/) were used to predict the conserved acetylation sites in the BCX‐XL mRNA CDS. (B) The relative levels of ac4C in BCL‐XL were tested by acRIP‐qPCR in each group of CFs. *n* = 6. (C) The relative levels of ac4C in BCL‐XL were tested by acRIP‐qPCR of each group in vivo. *n* = 6. (D) BCL‐XL mRNA levels were assessed by RT‐qPCR assay in each group of CFs. *n* = 3. (E) BCL‐XL protein levels were tested by western blot analysis in each group of CFs. *n* = 3. (F) BCL‐XL mRNA levels were assessed by RT‐qPCR assay of each group in vivo. *n* = 3. (G) BCL‐XL protein levels were tested by western blot analysis of each group in vivo. *n* = 3. (H) The mRNA stability was detected by RT‐qPCR in CFs with the addition of actinomycin D (5 μg/mL). *n* = 3. (I) Schematic illustration of the establishment of the two reformed luciferase reporter plasmids by inserting partial BCL‐XL mRNA sequences with wild‐type or mutant‐type. The luciferase activity was measured. *n* = 3.

In order to further elucidate the mechanism by which NAT10‐mediated RNA ac4C acetylation modification inhibits apoptosis in CFs, we conducted a review of relevant literature. Zhang et al. reported that NAT10 can acetylate BCL‐XL mRNA and inhibit apoptosis of multiple myeloma cells through the PI3K‐AKT pathway.^20^ Mechanistically, NAT10 strengthens the stability of BCL‐XL mRNA, a member of the Bcl‐2 protein family that promotes cell survival, facilitating protein translation and consequently inhibiting cellular apoptosis. Subsequently, we used the PACES tool, which predicted of ac4C sites in mRNA (http://rnanut.net/paces/), to predict conserved acetylation modification sites in the messenger RNA‐coding sequences of BCL‐XL (Figure 6A). This confirmed that NAT10 may indeed acetylate BCL‐XL mRNA to enhance its translational efficiency and inhibit apoptosis of CFs. Subsequently, acRIP‐qPCR assays performed both in vivo and in vitro robustly confirmed that the abundance of ac4C modification sites on BCL‐XL mRNA was significantly increased in TGF‐β1‐treated CFs or the myocardium of infarcted mice (Figure 6B,C). In vivo and in vitro consistently showed significant downregulation of BCL‐XL mRNA and protein levels following NAT10 acetylation inhibition by Remodelin (Figure 6D–G). This finding reinforces our conjecture. We treated CFs with actinomycin D (5 μg/mL) to examine the effect of Remodelin on the stability of BCL‐XL after inhibiting NAT10 acetylation through an RNA decay assay. The results obtained indicated that Remodelin could weaken BCL‐XL mRNA stability, and this effect could subsequently attenuate protein translation. (Figure 6H). To confirm that NAT10‐mediated ac4C modification affected BCL‐XL mRNA stability, we constructed recombinant luciferase reporter plasmids by inserting partial BCL‐XL mRNA sequences with wild‐type or mutated‐type (Figure 5I). The results showed that NAT10 inhibition failed to decrease the luciferase activity of the construct with mutation of BCL‐XL. Collectively, these results suggest that NAT10‐mediated RNA ac4C acetylation improves the stability of BCL‐XL mRNA and enhances its expression in CFs.

## 4 DISCUSSION

**FIGURE 7:**
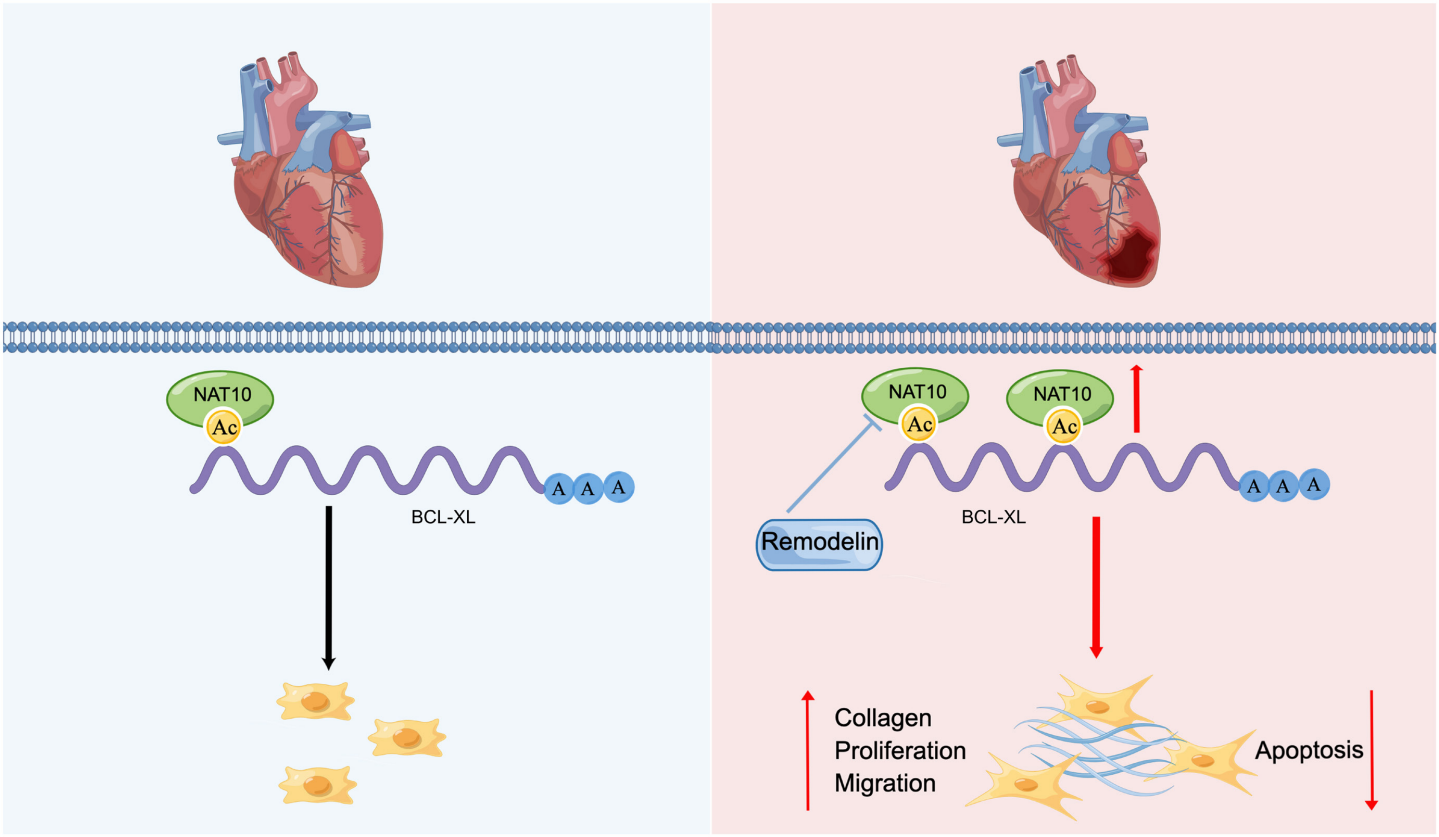
Mechanisms of inhibition of NAT10‐mediated BCL‐XL ac4C acetylation in regulating cardiac fibrosis.

In this study, it was observed that NAT10, the enzyme responsible for ac4C modification, exhibits a significant increase in expression in myocardial fibrosis tissues induced by myocardial infarction and CFs stimulated by TGF‐β1. The upregulation of NAT10 is associated with the promotion of fibroblast proliferation and differentiation into myofibroblasts, leading to increased collagen synthesis and deposition. On the contrary, the suppression of NAT10 mRNA ac4C modification through remodelin exhibits the potential to counteract these outcomes. Mechanistically, we have identified that Remodelin impairs the acetylation modification of BCL‐XL mRNA by inhibited NAT10, leading to decreased stability of the mRNA and subsequently downregulated protein expression. This downregulation activates Caspase3, which triggers apoptosis in CFs. Consequently, targeting the ac4C modification capacity of NAT10 may mitigate myocardial fibrosis following myocardial infarction in mice and potentially enhance cardiac function. This study represents a pioneering investigation into the crucial function of NAT10‐mediated mRNA acetylation in the modulation of myocardial fibrosis (Figure 7). According to the results of this study, it is anticipated that NAT10 will emerge as a novel therapeutic target for mitigating myocardial fibrosis following myocardial infarction.

CFs are the predominant effector cells involved in the initiation and progression of myocardial fibrosis, comprising two‐thirds of all cardiac cells, and are essential in the pathogenesis of myocardial fibrosis.^21^ Excessive deposition of ECM, particularly type I collagen, eventually leads to myocardial fibrosis and tissue function damage. In addition, CFs can secrete various ECM‐degrading proteases, such as matrix metalloproteinases (MMPs) together with tissue inhibitors of metalloproteinases (TIMPs), regulating ECM synthesis and degradation through multiple pathways.^22^, ^23^

RNA modification is an emerging field of epigenetics, playing a role in post‐transcriptional regulation in physiological and pathological processes. More than 170 kinds of RNA modifications have been discovered in humans, such as N1‐methyladenosine (m1A), N6‐methyladenosine (m6A), 5‐methylcytosine (m5C), as well as pseudouridine (Ψ).^24^, ^25^ Recently, multiple ac4C modifications have been observed in both human and yeast mRNAs.^26^ Arango et al. in 2018 revealed that ac4C was discovered in above 4000 regions of the human transcriptome.^7^ NAT10 is a key enzyme that catalyses the ac4C modification of mRNA.^27^ Subsequent research has demonstrated that NAT10 facilitates ac4C modification within the coding sequence (CDS). Suppression of NAT10 activity resulted in a decrease in ac4C modification levels, leading to reduced mRNA stability and translation efficiency, a phenomenon linked to its impact on codon selection.^7^ Dominissini et al. declared that NAT10‐mediated ac4C modification can also impact the interaction between codons and anticodons to regulate mRNA stability and translation efficiency.^28^ In contrast to the intricate regulatory mechanism involving the interplay of ‘writer,’ ‘eraser,’ and ‘reader’ proteins in the dynamic modulation of m6A methylation, the acetylation of ac4C lacks identified readers or erasers, with NAT10 serving as the sole writer responsible for catalysing RNA acetylation modification.^29^, ^30^

NAT10 is recognized as an oncogene, with alterations in its expression and localization observed in malignant tumour cells. These changes are believed to play a significant role in facilitating cancer cell invasion and migration and are closely associated with the epithelial‐mesenchymal transition (EMT) and chemoresistance of cancer cells. Such associations may have implications for the progression and prognosis of cancer patients.^9^, ^31^, ^32^ Wu et al. conducted an analysis on the modification of ac4C in the context of pulmonary fibrosis, utilizing lung epithelial and murine models to investigate potential mechanisms. The findings indicated a significant upregulation of NAT10 in pulmonary epithelial cells, leading to increased stability of TGF‐β1. Depletion of NAT10 provided notable protection against pulmonary EMT and fibrosis induced by PM2.5 exposure, while restoration of TGF‐β1 counteracted the protective effects of NAT10 inhibition.^33^ Nevertheless, the research on ac4C acetylation is unclear in myocardial fibres. Wang et al. identified a heart‐apoptosis‐associated piRNA (HAAPIR), which modulates cardiomyocyte apoptosis via targeting NAT10‐mediated ac4C acetylation of Tfec mRNA transcript.^12^ Their findings unravel that cardiomyocyte apoptosis is essential in the piRNA‐mediated ac4C acetylation mechanism. Shi et al. observed elevated levels of NAT10 expression and RNA ac4C in both in vitro and in vivo models of cardiac remodelling. Suppression and inhibition of NAT10 were shown to mitigate Ang II‐induced cardiomyocyte hypertrophy and cardiofibroblast activation. Additionally, the administration of the NAT10 inhibitor Remodelin was found to ameliorate cardiac functional deficits in mice undergoing transverse aortic constriction, by reducing cardiac fibrosis, hypertrophy, and inflammatory reactions, as well as modulating the expression of CD47 and ROCK2 (Rho associated coiled‐coil containing protein kinase 2).^34^ A recent investigation has revealed that the expression of the NAT10 gene is upregulated in mouse hearts subjected to ischemia/reperfusion (I/R) injury and in cardiomyocytes exposed to hypoxia followed by reoxygenation conditions.^35^ Notably, the excessive expression of NAT10 in cardiac tissue leads to the enhancement of cardiomyocyte ferroptosis, thereby exacerbating the damage caused by I/R injury. Conversely, targeted deletion of NAT10 specifically in cardiomyocytes or pharmacological suppression of NAT10 activity using Remodelin elicits opposing effects, mitigating the severity of the injury. Mechanistically, NAT10 promotes the ac4C modification of Mybbp1a protein, which stabilizes it and subsequently triggers a cascade of events involving the activation of p53 and the suppression of SLC7A11 gene transcription. SLC7A11 is a key anti‐ferroptotic gene, and its downregulation contributes to the observed exacerbation of ferroptosis in cardiomyocytes. Based on our research findings, we have discovered that the upregulation of NAT10 expression subsequent to myocardial infarction not only serves as a catalyst for cardiomyocyte apoptosis via the ferroptotic pathway but also exerts a suppressive influence on the apoptosis of CFs. This dual effect of NAT10 results in the stimulation of fibroblast proliferation and subsequent differentiation into myofibroblasts, contributing to a more intricate post‐infarction remodelling process.

BCL‐XL's well‐established role in modulating apoptosis has been extensively documented, encompassing both crucial embryonic developmental stages and various pathological conditions.^36^ Among the BCL‐2 anti‐apoptosis family of proteins, BCL‐XL is associated with BCL‐2, MCL‐1, BCL‐W, and BFL‐1, which act by binding to BH3 motifs on pro‐apoptotic proteins to regulate apoptosis.^37^ A recent investigation has investigated whether the anti‐apoptotic function was indeed responsible for BCL‐XL‐mediated metastasis. BCL‐XL executes its anti‐apoptotic function by binding to and inhibiting the pro‐apoptotic activity of Bax/Bak, which are otherwise poised to initiate the apoptotic cell death pathway.^38^ Our research directly observed that NAT10 regulates the proliferation of CFs and the differentiation into myofibroblasts through mediating BCL‐XL mRNA ac4C acetylation, ultimately affecting the pathophysiological process of myocardial fibrosis. Conversely, the suppression of NAT10‐mediated BCL‐XL ac4C acetylation led to the induction of caspase‐3‐dependent apoptosis in CFs, as well as the inhibition of CFs proliferation and the transition of fibroblasts into myofibroblasts induced by TGF‐β1. This work reveals the crucial role of NAT10‐mRNA ac4C acetylation in MI‐evoked myocardial fibrosis and TGF‐β1‐stimulated CFs proliferation and myofibroblasts. In light of these findings, our results demonstrate that NAT10‐regulated mRNA ac4C acetylation is a critical target for cardiac fibrosis treatment in clinical practice.

However, it is important to acknowledge the limitations of our study. Specifically, our observations were limited to the upregulation of NAT10 in cardiac fibroblasts treated with TGF‐β1 and in myocardial tissue post‐myocardial infarction at the cellular level. While inhibiting NAT10 acetylation function showed promise in improving cardiac fibroblast proliferation and myofibroblast transformation, our study did not explore the potential effects of NAT10 gene silencing. Additionally, the impact of NAT10 on myocardial cell apoptosis was not investigated. Furthermore, it is crucial to underscore that the correlations discerned in this research should not be misconstrued as definitive causal links. Additional investigations are imperative to delve into the underlying mechanisms of these observed associations and their potential consequences.

## 5 CONCLUSIONS

Our results found a novel mechanism by which NAT10 facilitates CFs proliferation and MFs transformation through increasing BCL‐XL mRNA ac4C acetylation manner.

## AUTHOR CONTRIBUTIONS

**Jun Li:** Conceptualization (equal); funding acquisition (equal); project administration (equal); writing – review and editing (equal). **Feierkaiti Yushanjiang:** Formal analysis (equal); methodology (equal); project administration (equal); software (equal). **Zhao Fang:** Formal analysis (equal); methodology (equal); software (equal); writing – original draft (equal). **Wan‐li Liu:** Conceptualization (equal); project administration (equal); writing – review and editing (equal).

## FUNDING INFORMATION

This work was supported by grants from Fundamental Research Funds for the Central Universities (2042021kf0132) and Natural Science Foundation of Hubei Province (no. 2024AFB216 and 2024AFB1049).

## CONFLICT OF INTEREST STATEMENT

The authors declare no competing interests.

## INSTITUTIONAL REVIEW BOARD STATEMENT

In this research, the animal procedures adhered to the standards of the Care and Use of Laboratory Animals and got approval from the Animal Use Committees of Renmin Hospital of Wuhan University (Approved ID: 20220104B, 1 February 2022).

## Data Availability

Data are available on reasonable request.
